# Assessment of early renal angina index for prediction of subsequent severe acute kidney injury during septic shock in children

**DOI:** 10.1186/s12882-020-02023-0

**Published:** 2020-08-20

**Authors:** Linlin Huang, Ting Shi, Wei Quan, Weiming Li, Lili Zhang, Xueping Liu, Saihu Huang, Ying Li, Xiaozhong Li

**Affiliations:** 1 Pediatric Intensive Care Unit, Children’s Hospital of Soochow University, Suzhou, China; 2 Department of Epidemiology, Children’s Hospital of Soochow University, Suzhou, China; 3 Department of Nephrology and Immunology, Children’s Hospital of Soochow University, Suzhou, China; 4 Department of Anesthesiology, Children’s Hospital of Soochow University, Suzhou, China

**Keywords:** Acute kidney injury, Renal angina index, Septic shock, Predictor

## Abstract

**Background:**

Acute kidney injury (AKI) is independently related to the adverse outcome of septic shock, but it lacks effective early predictors. Renal anginal index (RAI) was used to predict subsequent severe AKI (AKIs) in critically ill patients. The application of RAI in children with septic shock has not been reported. This study aims to evaluate the efficacy of early RAI in predicting subsequent AKIs within 3 days after PICU admission in children with septic shock by comparing with early fluid overload (FO) and early creatinine elevation.

**Methods:**

Sixty-six children admitted to PICU aged 1 month to 16 years old, with septic shock from January 2016 to December 2019 were analyzed retrospectively. According to the 2012 Kidney Disease Improving Global outcomes (KDIGO) criteria, AKIs was defined by the KDIGO stage ≥2 within 3 days after PICU admission. Early RAI positive (RAI+) was defined as RAI ≥ 8 within 12 h of PICU admission. Any elevation of serum creatinine (SCr) over baseline within 12 h after PICU admission was denoted as “Early SCr > base”. Early FO positive (FO+) was defined as FO > 10% within 24 h of PICU admission.

**Results:**

Of 66 eligible cases, the ratio of early RAI+, early SCr > base, early FO+ was 57.57, 59.09 and 16.67% respectively. The incidence of AKIs in early RAI+ group (78.94%) was higher than that in early RAI- group (21.42%) (*p* = 0.04), and there was no significant difference compared with the early FO+ group (71.79%) and early SCr > base group (81.82%) (*P* > 0.05). After adjustment for confounders, early RAI+ was independently associated with the occurrence of AKIs within 3 days (OR 10.04, 95%CI 2.39–42.21, *p* < 0.01). The value of early RAI+ (AUC = 0.78) to identify patients at high risk of AKIs was superior to that of early SCr > base (AUC = 0.70) and early FO+ (AUC = 0.58). A combination of serum lactate with early RAI+ improved the predictive performance for assessing AKIs (AUC = 0.83).

**Conclusions:**

Early RAI could be used as a more convenient and effective index to predict the risk of AKIs in children with septic shock within 3 days. Early RAI+ combined with serum lactate improved the predictive performance for assessing AKIs.

## Background

Acute kidney injury (AKI) often occurs early after PICU admission in patients with septic shock and the incidence is 59–72% [1–3]. During septic shock, decreased cortical renal perfusion was observed by renal contrast-enhanced ultrasound, which is supposed to be part of the cause of AKIs [4]. AKIs is independently associated with increased morbidity and mortality [1]. Current management guidelines for patients with AKI recommend that early recognition of AKI risks and augmentation of supportive care will limit AKI progression [5].

According to the 2012 KDIGO criteria, the definition of AKI depends on serum creatinine and urine volume. The accuracy of any creatinine-based or urine volume-based classification is likely to be affected by age, pre-existing sarcopenia, catabolism and fluid intake and diuretic, respectively [5]. Due to the uncertainty of serum creatinine and urine volume, the diagnosis of AKI is often delayed, which creates great obstacles for effective early intervention. One of the ways to solve this problem is to combine other clinical indicators to alleviate the uncertainty of creatinine and urine volume in judging renal function.

The concept of renal angina index (RAI) combines risk factors and early signs of injury, intending to stratify patients at risk to avoid subsequent AKIs [6]. According to reports, RAI can improve accuracy for prediction of AKIs in critically ill children and young adults [7–9]. However, there are no reports of RAI used to predict AKI in patients with septic shock. Our aim of this study is to evaluate the efficacy of early RAI in predicting subsequent AKIs within 3 days after PICU in children with septic shock by comparing with early FO and early creatinine elevation.

## Methods

### Study design and participants

This study was conducted at the children’s hospital of Soochow University, a tertiary pediatric hospital from China. The study was approved by the Ethics Committee of Children’s Hospital of Soochow University, and granted a waiver of informed consent. All hospitalized patients aged 1 month to 16 years old who were diagnosed with septic shock in PICU from January 2016 to December 2019 were retrospectively reviewed. Deep A et al. found that most patients developed AKI within the first 48 h of PICU admission [1]. So, patients with previously known kidney diseases, patients with hospital stay less than 48 h, patients who developed AKI 3 days after PICU admission and AKIs within 12 h after PICU admission were excluded.

### Data collection

Electronic records were reviewed. Data were collected on demographics, medical history, current illness, laboratory variables, pediatric scores of critical illness (PRISM III score and PELOD-2 score) at the first day of PICU admission. Data was also recorded as follow: fluid balance, PICU and hospital lengths of stay, need for mechanical ventilation (MV) and renal replacement therapy (RRT), early vasoactive support (early VS), AKI stage within 3 days after PICU admission and follow-up.

### Definition

Baseline serum creatinine (SCr) was considered as the lowest SCr in the 3 months before admission. When baseline SCr was unavailable, a baseline serum creatinine was calculated by the Schwartz formula with an estimated glomerular filtration (eGFR) of 120 ml/min/1.73m^2^ [10]. For both RAI and elevated creatinine determination, maximum SCr in the first 12 h of PICU admission was used. Elevated SCr within 12 h after PICU admission was denoted as “early SCr > base”.

Early RAI was defined as the product of risk group score and renal injury score, calculated base on clinical data in the first 12 h of PICU admission. We adopted the RAI model proposed by Basu et al. in 2018 [8]. Risk strata include PICU admission, solid organ or stem-cell transplantation, MV and vasoactive support. Injury strata contains creatinine elevation and FO. It is worth noting that small changes in serum creatinine of less than 1.5 times are concerned, which is different from the definition of creatinine in the KDIGO criteria on AKI. The index ≥8 was considered RAI+, and the score < 8 was defined as RAI-. The method of calculation was shown in Table 1 [8].

**Table 1:** The Renal Angina Index

**Risk strata**		Score	
Admission to PICU		1	
Solid organ Or Stem-cell transplantation		3	
Mechanical ventilation Or Vasoactive support or both		5	
**Injury strata**			**Risk × injury scores:1–40**
**SCr/base**	**%FO accumulation**		
Decreased or no change	< 5%	1	
> 1 × −1.49×	5–10%	2	
1.5 × −1.99×	10–15%	4	
≥2×	> 15%	8	

Renal angina index (RAI) was calculated basing on the clinical data within the first 12 h after PICU admission. And RAI ≥8 was defined as RAI positive

*PICU* pediatric intensive care unit, *SCr* serum creatinine, *FO* fluid overload

Early fluid overload (FO) was calculated 24 h after admission with the formula as follow [11]: percentage of FO (%) = [total fluid in(L)-total fluid out(L)]/ admission body weight (kg) × 100. It has been reported that FO > 10% is related to higher mortality in children with septic shock [12]. FO > 10% was defined as FO+, otherwise it was FO-.

AKI was defined and classified according to the 2012 KDIGO criteria. AKIs was defined by the KDIGAO stage ≥2 within 3 days after PICU admission. Patients with AKIs (KDIGO Stage 2–3) were divided into AKIs group, otherwise non-AKIs group.

Septic shock was defined as sepsis-induced hypotension persisting despite adequate fluid resuscitation [13]. Vasoactive support is required to maintain an appropriate mean arterial pressure in patients with septic shock. The need for vasoactive support within 12 h of PICU hospitalization was defined as early VS.

### Statistical analysis

Continuous variables with symmetric distribution were presented as the mean ± standard deviation (SD), while asymmetric distribution with median and interquartile (IQR). Categorical variables were expressed as frequency and percentage. Continuous variables with a normal distribution were compared between groups using Student’s t-test, and with a nonnormal distribution using the Wilcoxon ranked test. Statistical significance of differences between categorical variables was evaluated using the chi-squared test. Logistic regression was used to identify predictors for subsequent AKIs within 3 days after PICU admission. The efficiency of risk factors for predicting AKIs was evaluated by the area under the curve (AUC) of the receiver operating characteristic (ROC) curve. All the analyses were conducted in IBM-SPSS version 25(IBM corporation, Armonk, New York, USA), and *p*<0.05 from 2-sided tests was considered statistically significant.

## Results

### Patients characteristics

**Fig. 1:**
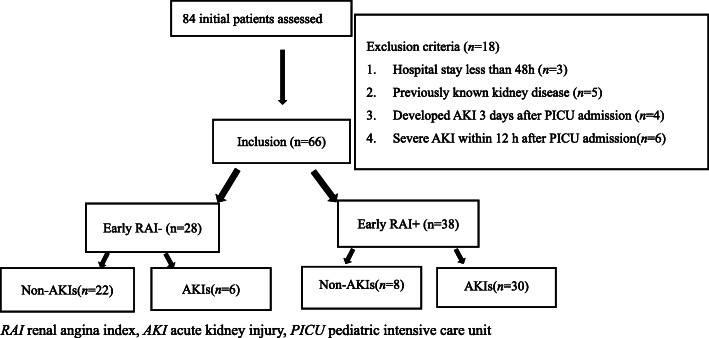
Flow of participants in the study

Of the total 84 patients with septic shock admitted to PICU during the period, 66 patients met the requirement of this study and there was no missing data. (Fig. 1). Eighteen patients were excluded for the following reasons: hospital stay less than 48 h *(n* = 3), previously known kidney disease (*n* = 5), developed AKI 3 days after admission(*n* = 4), AKIs within 12 h of PICU admission (*n* = 6). Of all the eligible cases, age was 58.00 (8.50–131.75) months, 44(66.70%) were male, 36(54.50%) developed AKIs within 3 days after PICU admission, 37 (56.60%) needed MV, 16(24.24%) needed RRT, 38(57.60%) needed early VS, 41(62.10%) had underlying diseases and 27(40.90%) died. The most common infection foci were respiratory tract (54.54%), followed by gastrointestinal infections (28.79%). According to the 2012 KDIGO criteria, 53(80.30%) patients developed AKI: 17 (32.08%) stage1, 5 (9.43%) stage2, and 31 (58.49%) stage3.

### Patients who died had a higher incidence of AKIs

The incidence of AKIs in the non-survivors (92.59%) was higher than that in the survivors (28.21%) (*p* < 0.01). The proportion of patients without AKI in non-survivors (3.70%) was lower than that in the survivors (30.77%) (*p* = 0.02). (Table 2).

**Table 2:** Incidence of AKI within 3 days after PICU admission between survivors and non-survivors

Variable	Survivors(*n* = 39)	Non-survivors(*n* = 27)	*p*-value
Non-AKI, *n* (%)	12 (30.77)	1 (3.70)	0.02
Stage 1, *n* (%)	16 (41.03)	1 (3.70)	0.01
Stage2, *n* (%)	4 (10.25)	1 (3.70)	0.32
Stage3, *n* (%)	7 (17.95)	24 (88.90)	<0.01
Severe AKI, *n* (%)	11 (28.21)	25 (92.59)	<0.01
Early RAI+, *n* (%)	16 (41.02)	22 (81.48)	<0.01
Early FO+, *n* (%)	3 (7.69)	8 (29.63)	0.02
Early SCr > base, *n* (%)	20 (51.28)	19 (70.37)	0.12

*AKI* acute kidney injury, *RAI* renal angina index, *FO* fluid overload, *SCr* serum creatinine

### The ratio of early RAI+, early SCr > base, early FO+ was higher in AKIs group

The age (57.00 (2.00–137.00) months vs. 59.00 (16.50–124.00) months, *p* = 0.68), percentage of males (66.67% vs. 66.67%, *p* = 1), and underlying disease (50.00% vs. 72.22%, *p* = 0.06) was similar between non-AKIs group and AKIs group. Compared with non-AKIs group, the baseline laboratory results such as serum lactate (4.00 (2.80–9.10) mmol/L vs. 2.80 (1.65–3.87) mmol/L, *p* = 0.01), active partial thromboplastin time (55.85 (48.15–76.17) s vs. 46.20 (37.05–51.77) s, *p* < 0.01) were increased in AKIs group, and serum albumin (30.38 ± 7.35 g/L vs. 33.92 ± 6.09 g/L, *p* = 0.04), platelet count (32.00 (15.00–83.25) × 10^9^/L vs. 225.50 (30.75–316.00) × 10^9^/L, *p* < 0.01) decreased. The AKIs group had higher PRISM III score (14.00 (12.00–18.00) vs. 6.00 (3.00–12.00), *p* < 0.01), PELOD-2 score (7.00 (4.00–9.75)vs. 3.00 (2.00–5.25), *p* < 0.01); increased utilization of RRT (36.11% vs.10.00%, *p* = 0.01), MV (77.78% vs.30.00%, *p* < 0.01), early VS (80.56% vs. 30.00%, *p* < 0.01); and increased mortality (69.44% vs. 6.67%, *p* < 0.01). Three patients in non-AKIs group had RRT due to fluid overload. The ratio of early SCr > base (77.78% vs. 36.67%, *p* < 0.01), early RAI+ (83.33% vs. 26.67%, *p* < 0.01), and early FO+ (25.00% vs. 6.67%, *p* = 0.04) was higher in AKIs group. (Table 3).

**Table 3:** Baseline laboratory and patient characteristics between Non-AKIs and AKIs

Variable	Non-AKIs(*n* = 30)	AKIs(*n* = 36)	*p*-value
Age in months	57.00 (2.00–137.00)	59.00 (16.50–124.00)	0.68
Male, *n* (%)	20 (66.67)	24 (66.67)	1.00
Underlying disease, *n* (%)	15 (50.00)	26 (72.22)	0.06
Early SCr > base, *n* (%)	11 (36.67)	28 (77.78)	< 0.01
Early RAI+, *n* (%)	8 (26.67)	30 (83.33)	< 0.01
Early FO+, *n* (%)	2 (6.67)	9 (25.00)	0.04
Baseline laboratory Results			
Serum albumin (g/L)	33.92 ± 6.09	30.38 ± 7.35	0.04
Serum chloride (mmol/L)	102.17 ± 4.79	102.46 ± 10.66	0.89
Serum lactate (mmol/L)	2.80 (1.65–3.87)	4.00 (2.80–9.10)	0.01
Active partial thromboplastin time (s)	46.20 (37.05–51.77)	55.85 (48.15–76.17)	< 0.01
Thromboplastin time (s)	16.90 (14.42–18.85)	18.85 (16.02–24.77)	0.01
Leukocyte count (10^9^/L)	10.61 ± 18.35	13.68 ± 22.46	0.55
Hemoglobin (g/L)	94.43 ± 29.80	85.75 ± 29.25	0.24
Platelet count (×10^9^/L)	225.50 (30.75–316.00)	32.00 (15.00–83.25)	< 0.01
Severity of Illness			
PRISM III score	6.00 (3.00–12.00)	14.00 (12.00–18.00)	< 0.01
PELOD-2 score	3.00 (2.00–5.25)	7.00 (4.00–9.75)	< 0.01
Mechanical ventilation, *n* (%)	9 (30.00)	28 (77.78)	< 0.01
Renal replacement therapy, *n* (%)	3 (10.00)	13 (36.11)	0.01
Early VS, *n* (%)	9 (30.00)	29 (80.56)	< 0.01
Outcomes			
ICU LOS (d)	8.50 (4.00–10.00)	5.50 (4.50–8.00)	0.06
Hospital LOS (d)	19.50 (17.00–28.25)	17.50 (5.00–24.00)	0.03
Mortality *n* (%)	2 (6.67)	25 (69.44)	< 0.01

*AKI* acute kidney injury, *RAI* renal angina index, *FO* fluid overload, *SCr* serum creatinine, *PRISM III* pediatric risk of mortality III, *LOS* length of stay, *PELOD-2* pediatric logistic organ dysfunction-2, *PICU* pediatric intensive care unit, VS vasoactive support

### The patients with early RAI+ had higher incidence of AKIs and poor outcomes

Early RAI+ occurred in 38/66 (57.57%) of patients. Compared to early RA- group, early RA+ group had higher PRISM III score (14.00 (12.00–18.25) vs. 6.50 (3.00–9.00), *p* < 0.01) and PELOD-2 score (7.00 (4.00–9.00) vs.3.00 (2.00–4.00), *p* < 0.01). Early RAI+ group was associated with a higher incidence of MV (73.68% vs. 32.14%, *p* < 0.01), RRT (34.21% vs.10.71%, *p* = 0.02), early VS (73.68% vs. 35.71%, *p* = 0.02), and increased mortality (57.89% vs. 17.85%, *p* < 0.01). The proportion of AKIs in early RAI+ group (78.94%) was higher than that in early RAI- group (21.42%) (*p* = 0.04). (Table 4).

**Table 4:** The severity of illness and outcomes between early RAI- group and early RAI+ group

Variable	Early RAI-(*n* = 28)	Early RAI+(*n* = 38)	*p*-value
Age in months	71.00 (5.50–134.00)	57.00 (10.00–132.00)	0.88
Male, *n* (%)	20 (71.42)	24 (63.15)	0.48
Underlying disease, *n* (%)	15 (53.57)	26 (68.42)	0.21
Early SCr > base, *n* (%)	9 (32.14)	30 (78.94)	< 0.01
Early FO+, *n* (%)	3 (10.71)	8 (21.05)	0.26
Severity of Illness			
PRISM III score	6.50 (3.00–9.00)	14.00 (12.00–18.25)	< 0.01
PELOD-2 score	3.00 (2.00–4.00)	7.00 (4.00–9.00)	< 0.01
Mechanical ventilation, *n* (%)	9 (32.14)	28 (73.68)	< 0.01
Renal replacement therapy, *n* (%)	3 (10.71)	13 (34.21)	0.02
Early VS, *n* (%)	10 (35.71)	28 (73.68)	0.02
AKI incidence within 1 week after admission to PICU			
NO AKI, *n* (%)	12 (42.85)	1 (2.63)	< 0.01
Stage 1, *n* (%)	10 (35.71)	7 (18.43)	0.11
Stage2, *n* (%)	2 (7.14)	3 (7.89)	0.91
Stage3, *n* (%)	4 (14.28)	27 (73.68)	< 0.01
Severe AKI, *n* (%)	6 (21.42)	30 (78.94)	0.04
Outcomes			
ICU LOS (d)	7.50 (4.00–10.75)	5.50 (3.50–9.00)	0.11
Hospital LOS (d)	20.50 (16.50–27.75)	17.00 (8.00–26.50)	0.14
Mortality n (%)	5 (17.85)	22 (57.89)	< 0.01

*AKI* acute kidney injury, *RAI* renal angina index, *FO* fluid overload, *SCr* serum creatinine, *PRISM III* pediatric risk of mortality III, *LOS* length of stay, *PELOD-2* pediatric logistic organ dysfunction-2, *PICU* pediatric intensive care unit, VS vasoactive support

### The incidence of AKIs in early SCr > base group and early FO+ group was similar to that in early RAI+ group

The incidence of early RAI+, early SCr > base and early FO+ was 38/66 (57.57%), 39/66 (59.09%) and 11/66(16.67%), respectively. Table 5 showed that PRISM III score (14.00 (12.00–18.25) vs. 13.00 (9.00–17.00) vs. 17.00 (12.00–19.00), *p* = 0.43) and PELOD-2 score (7.00 (4.00–9.00) vs. 5.00 (3.00–10.00) vs. 6.00 (4.00–9.00), *p* = 0.37) were similar in three groups. The proportion of MV (73.68% vs. 64.10% vs. 81.82%, *p* = 0.44), RRT (34.21% vs. 30.76% vs.27.27%, *p* = 0.89), early VS (73.68% vs. 66.66% vs. 72.73%, *p* = 0.78) and mortality (57.68% vs. 48.72% vs. 72.73%, *p* = 0.34) did not demonstrate a significant difference among groups. Early RAI+ group, early SCr > base group, and early FO+ group demonstrated a similar ratio of AKIs (78.94% vs. 71.79% vs. 81.82%, *p* = 0.68).

**Table 5:** Early RAI+ versus early SCr > base versus early FO+

Variable	Early RAI+ (*n* = 38)	Early SCr > base(*n* = 39)	Early FO+ (*n* = 11)	*p*-value
Age in months	57.00 (10.00–132.00)	51.50 (8.50–132.00)	60.00 (21.50–125.00)	0.98
Male, *n* (%)	24 (63.15)	25 (64.10)	8 (72.73)	0.83
Underlying disease, *n* (%)	26 (68.42)	24 (61.53)	8 (72.73)	0.71
Severity of Illness				
PRISM III score	14.00 (12.00–18.25)	13.00 (9.00–17.00)	17.00 (12.00–19.00)	0.43
PELOD-2 score	7.00 (4.00–9.00)	5.00 (3.00–10.00)	6.00 (4.00–9.00)	0.37
Mechanical ventilation, *n* (%)	28 (73.68)	25 (64.10)	9 (81.82)	0.44
Renal replacement therapy, *n* (%)	13 (34.21)	12 (30.76)	3 (27.27)	0.89
Early VS, *n* (%)	28 (73.68)	26 (66.66)	8 (72.73)	0.78
AKI incidence within 1 week after admission to PICU				
NO AKI, *n* (%)	1 (2.63)	1 (2.57)	2 (18.18)	0.06
Stage 1, *n* (%)	7 (18.43)	10 (25.64)	0 (0.00)	0.16
Stage2, *n* (%)	3 (7.89)	3 (7.69)	1 (9.09)	0.98
Stage3, *n* (%)	27 (71.05)	25 (64.10)	8 (72.73)	0.76
Severe AKI, *n* (%)	30 (78.94)	28 (71.79)	9 (81.82)	0.68
Outcomes				
ICU LOS (d)	5.50 (3.50–9.00)	5.00 (4.00–11.00)	4.00 (3.5–7.00)	0.94
Hospital LOS (d)	17.00 (8.00–26.50)	19.00 (9.00–24.00)	19.00 (1.00–37.00)	0.89
Mortality *n* (%)	22 (57.89)	19 (48.72)	8 (72.73)	0.34

*AKI* acute kidney injury, *RAI* renal angina index, *FO* fluid overload, *SCr* serum creatinine, *PRISM III* pediatric risk of mortality III, *LOS* length of stay, *PELOD-2* pediatric logistic organ dysfunction-2, *PICU* pediatric intensive care unit, *VS* vasoactive support

### The prediction value of early RAI+ for AKIs in children with septic shock was significantly higher than that of early FO+ and early SCr > base

**Fig. 2:**
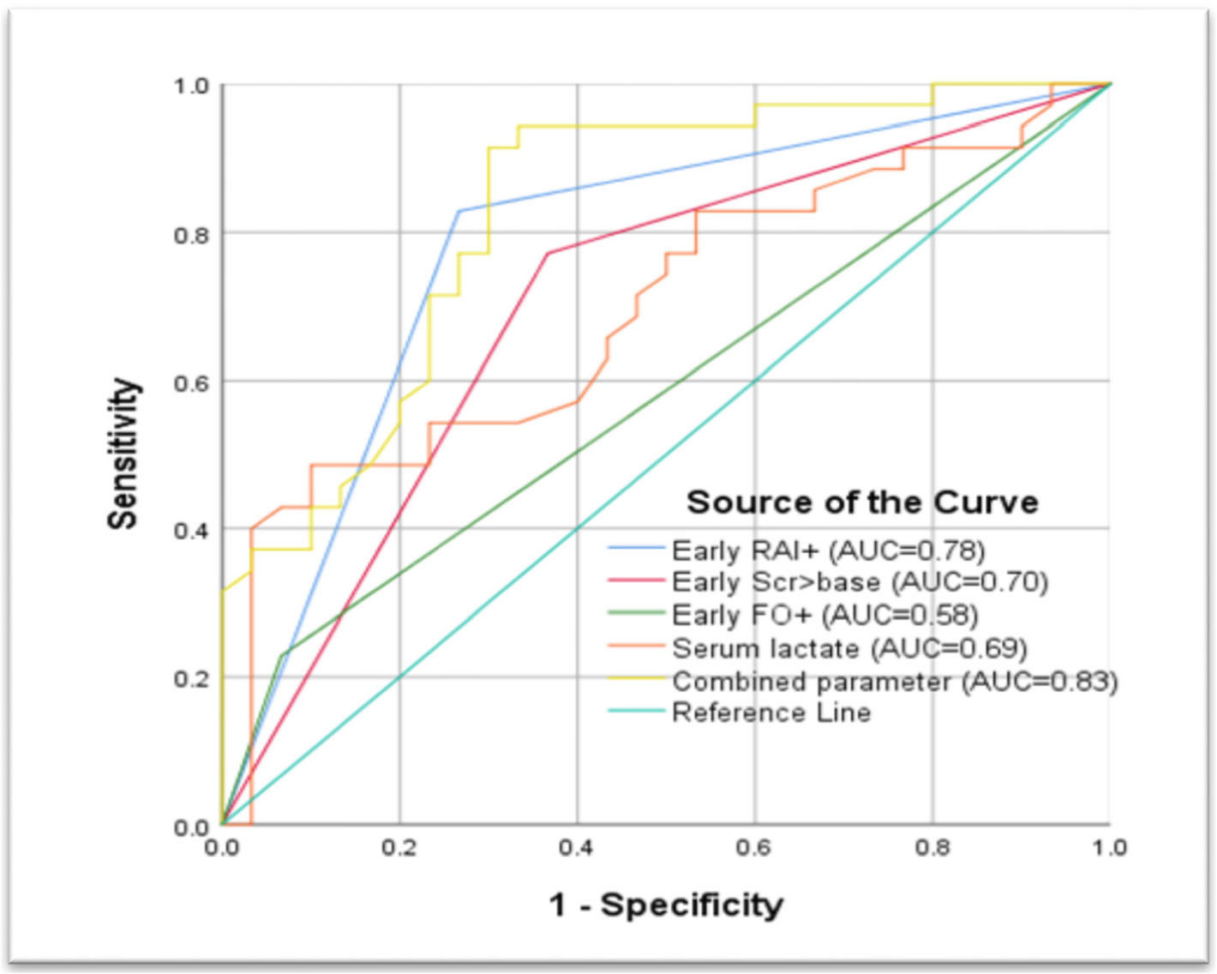
Receiver operating characteristic (ROC) curves for prediction of severe acute kidney injury. *RAI* renal angina index, *FO* fluid overload, *SCr* serum creatinine, *AUC* the area under the curve. Combined parameter: early RAI+ and serum lactate

Univariate regression analysis done to evaluate the effect of individual parameters showed that early RAI+, early SCr > base, PRISM III score, PELOD-2 score, MV, early VS, serum albumin, serum lactate were significantly associated with the occurrence of AKIs within 3 days after admission to PICU (*p* < 0.05). Multivariate analysis showed that only early RAI+ (OR 10.04, 95%*CI* 2.39–42.21, *p* < 0.01), early VS (OR 9.08, 95%*CI* 2.10–39.22, *p* < 0.01), serum lactate (OR 1.24, 95%*CI* 1.03–1.50,*p* = 0.02) were independently associated with the occurrence of AKIs within 3 days (Table 6). The value of early RAI+ (AUC 0.78, 95%*CI* 0.65–0.89, *p* < 0.01) to identify patients at high risk of AKIs was superior to that of early SCr > base (AUC 0.70, 95%*CI* 0.56–0.83, *p* < 0.01) and early FO+ (AUC 0.58, 95%*CI* 0.44–0.72, *p* = 0.24). (Fig. 2).

**Table 6:** Logistic regression analysis for severe acute kidney injury

variable	Univariate analysis			Multivariate analysis		
	OR	95%CI	*p*-value	OR	95%CI	*p*-value
Early RAI+	13.75	4.17–45.33	< 0.01	10.04	2.39–42.21	< 0.01
Early SCr > base	6.04	2.05–17.82	< 0.01			
Early FO+	4.66	0.92–23.62	0.06			
PRISM III score	1.26	1.12–1.42	< 0.01			
PELOD-2 score	1.47	1.21–1.80	< 0.01			
Underlying disease	2.60	0.93–7.22	0.06			
MV	8.16	2.69–24.72	< 0.01			
Early VS	11.27	3.48–36.54	< 0.01	9.08	2.10–39.22	< 0.01
Serum albumin	0.92	0.85–0.998	0.04			
Serum chloride	1.00	0.94–1.06	0.88			
Serum lactate	1.24	1.04–1.48	0.01	1.24	1.03–1.50	0.02

*RAI* renal angina index, *FO* fluid overload, *SCr* serum creatinine, *PRISM III* pediatric risk of mortality III, *LOS* length of stay, *PELOD-2* pediatric logistic organ dysfunction-2, *CI* confidence interval, *OR* odds ratio, *MV* mechanical ventilation, *VS* vasoactive support

### Early RAI+ combined with serum lactate improved the predictive performance for assessing AKIs

Logistic analysis showed that early RAI+ and serum lactate were associated with incidence of AKIs. AUC of serum lactate for predicting AKIs was 0.69 (95% *CI* 0.57–0.82, *p* = 0.01) with optimal cut-off 4.80 mmol/L. A combination of serum lactate with early RAI+ improved the predictive performance for assessing AKIs (AUC 0.83, 95% *CI* 0.73–0.93, *p* < 0.01) (Fig. 2).

## Discussion

Our retrospective study revealed that early RAI measured within the first 12 h of PICU admission was able to predict the development of secondary AKIs in children with septic shock and the value of early RAI+ in distinguishing the risk of AKIs was better than early SCr > base and early FO+.

AKI is one of the common complications of septic shock. The mechanism of AKI in septic shock is complex. Hypoperfusion is the direct cause of AKI caused by septic shock. Disturbance of other system functions caused by hypoperfusion, such as abnormal blood coagulation, can also induce AKI. Compared with the non-AKIs group, patients with AKIs in this study had higher serum lactate concentration and active partial thromboplastin time, which reflected the status of tissue perfusion and coagulation, respectively. Some treatments such as MV [14] and vasoactive support [15] are related to AKI, and this study also found that the proportion of MV and early VS in the AKIs group was higher than that in the non-AKIs group. AKIs is independently associated with poor outcomes in children with septic shock [16]. Early prediction of AKIs can improve the prognosis of critical patients. Creatinine elevation and urine output is the most widely used and convenient index to evaluate renal function. However, they have limitations. Therefore, it is urgent to find novel evaluation indexes of AKI.

The concept of renal angina index (RAI) combines risk factors and early signs of injury, which has been validated and improved to predict AKIs. RAI ≥ 8 is defined as RAI+, and it has been proved to be associated with AKIs and worse prognosis in critically ill patients [7, 8, 17]. The incidence of AKIs in early RAI+ group was higher than that in early RAI- group in our study. We also observed that the patients in the early RAI+ group were more serious with higher mortality and needed more MV, RRT and early VS. It has been reported that the incorporation of other indicators of kidney injury can further increase the predictive power of RAI. Fluid overload [18], urinary neutrophil gelatinase-associated lipocalin [19] and urinary L-type fatty acid-binding protein [20] combined with RAI have been shown to optimize AKI prediction in critically ill patients.

As a component of RAI, FO is also used as an indicator of disease severity. FO represents the states of fluid balance, which comprehensively reflects fluid intake, cardiovascular function and renal function. Kelm DJ et al. suggested that persistent FO was associated with increased use of fluid-related medical intervention and hospital mortality in patients with severe sepsis and septic shock [21]. FO for more than 2 days increased the risk of kidney failure in children with septic shock [22]. Another study has shown that FO > 10% is related to a higher mortality [12]. In this study, the incidence of early FO+ was higher in non-survivors and AKIs group than that in survivors and non-AKIs group, respectively. However, our results showed that early FO+ was not a sensitive indicator for predicting AKIs in patients with septic shock.

Previous reports on predicting AKIs by RAI were mainly aimed at critical patients in ICU, but little attention focused on patients with septic shock alone. In this study, the feasibility of predicting AKIs by early RAI+ was discussed by comparing with early creatinine elevation and early FO+. The incidences of early RAI+, early SCr > base and early FO+ were 57.57, 59.09 and 16.67%, respectively. We found that early RAI+ demonstrated better prediction for AKIs than early FO+ and early SCr > base in children with septic shock. The performance of early RAI+ and early SCr > base in predicting AKIs in our study were parallel with that in critically ill patients [8, 9]. The reason why RAI is better than FO and SCr > base in predicting AKIs maybe that RAI combines early signs of renal injury with the risk factors. Early RAI+ was also associated with higher mortality, MV, RRT and early VS in this study. Therefore, early renal protection intervention should be necessary for patients with RAI+.

In addition to early RAI+, serum lactate was also independently associated with AKIs within 3 days of PICU admission in our study. Inclusion of serum lactate with early RAI+ improved the predictive performance for assessing AKIs (AUC:0.83). Elevated serum lactate indicates microcirculation disorder, and can also reflect the status of renal microcirculation, which could be used to further improve the strata of renal injury in the RAI score. The RAI model is still being improved to facilitate operation and improve detection accuracy. Hanson HR et al. reported an acute RAI model for ruling out the development of in-hospital AKI at emergency department [23].

Our study has several limitations. First, the retrospective nature of this study was its greatest limitation because the results depended on the accuracy and completeness of patient records. Second, baseline serum creatinine was not available in some patients, which was estimated by the Schwartz formula with an estimated glomerular filtration (eGFR) of 120 ml/min/1.73m^2^. Third, it is a small sample study, further randomized and controlled clinical trials are needed to determine the efficacy of RAI in predicting AKIs in children with septic shock.

## Conclusions

The incidence of AKIs in patients with early RAI+ reached 78.94%. Patients with early RAI+ were in serious condition with higher mortality, needed more MV, RRT and early VS. Compared with context-free changes in early SCr and early FO, early RAI+ could be used as a more convenient and effective index to predict the risk of AKIs in children with septic shock. Inclusion of serum lactate with early RAI+ improved the predictive performance for assessing AKIs.

## Data Availability

All data generated or analyzed during this study are included in this published article.

## Abbreviations

AKI: Acute kidney injury
AKIs: Severe acute kidney injury
RAI: Renal anginal index
FO: Fluid overload
eGFR: Estimated glomerular filtration
SCr: Serum creatinine
BUN: Serum urea nitrogen
UA: Uric acid
VS: Vasoactive support
WBC: White blood cell
CRP: C-reactive protein
PCT: Procalcitonin
RRT: Renal replacement therapy
MV: Mechanical ventilation
PRISM III: Pediatric risk of mortality III
PELOD-2: Pediatric logistic organ dysfunction-2
PICU: Pediatric intensive care unit
